# On the Elephantiasis Græcorum, or Spedalskhed of Norway, &c.

**Published:** 1844-10

**Authors:** 


### ART. VI.

1. *Om den spedalske Sygdom* (*Elephantiasis Græcorum.*) Af Berglaege C. W. BOECK.

*On the Elephantiasis Græcorum, or Spedalskhed of Norway, &c.* By C. W. BOECK, Physician to the Mines at Kongsberg.

2. *Iagttagelser om Spedalske i St. Jörgens Hospital i Bergen i* 1841. Ved Corpslaege D. C. DANIELSEN i Bergen.

*Observations on the Patients affected with Lepra in St. George's Hospital in Bergen.* By Dr. DANIELSEN. (*Both Essays are published in the 'Norsk Magazin for Laegevidenskaben.'—Christiania,* 1842.)

THE Norwegian government have now for some years directed the attention of the medical profession in that country to a thorough investigation of the diseases which appear to be almost peculiar to the high northern regions. Not contented with the consideration of these disorders at home, several enterprising and public-spirited individuals have undertaken long and arduous journeys to the different parts of Europe where maladies analogous to those of Norway are reported to prevail. The result of one of these scientific expeditions—for such we may truly call them—is here presented in the report of Dr. Boeck, and the admirable essay by Dr. Danielsen, Physician to St. George's Hospital at Bergen, is another proof of the interest that is now excited in Norway regarding these two diseases, the radesyge and the spedalskhed or leprosy. Nor is the study of these maladies—though they are now fortunately confined to a very few isolated spots in Europe—without interest to the practitioner in more favoured lands. In a historical point of view, they are of the utmost importance, as there seems now no doubt of the fact, that the ancient leprosy, which formerly prevailed to so fearful an extent in Britain, is still to be found under the name of the spedalskhed, liktraa, and radesyge in the remote regions of Iceland and of Norway. In a former volume of this Review (April 1842,) we gave a detailed analysis of Dr. Hjort's description of the last-named malady. Dr. Hjort was sent, in 1838, to investigate the disorders analogous to radesyge in the southern parts of Europe; and in like manner his talented colleague, Dr. Boeck, has now recorded his researches concerning the prevalence of the spedalskhed on the western coast of Norway, and his experience of the same disease, as it exists on the shores of the Mediterranean and in the Grecian Archipelago. Dr. Danielsen's paper is not an essay upon the disease, but merely professes to be a report of the patients treated in St. George's Hospital at Bergen during the year 1841. The number of these patients amounted to 155, of which 79 were females and 76 were males. The deaths during the year were 16 in number; and 13 post-mortem examinations were made, which last, we believe, are the first records of the state of the internal organs in this disease.

Both Dr. Danielsen and Dr. Boeck admit two varieties of elephantiasis Græcorum, viz. elephantiasis anaisthetos (*αναισθητος*,) or glabra,* and elephantiasis tuberculosa. We shall endeavour to lay before the reader a succint history of both these forms of spedalskhed, as they are described in the two works at the head of this article.

First form. *Elephantiasis anaisthetos seu glabra.* This form of the malady is occasionally preceded by a certain train of symptoms, such as anorexia, general debility, and a tendency to sleep ; or occasionally the patients experience a sensation of intense cold, which may pervade the whole body or be confined merely to a single member. But of all indications of the speedy invasion of the disease, Dr. Boeck regards the appearance of *pemphigus*, chiefly upon the elbows or knees, as one of the most certain. The patients so affected generally affirm that they went to bed in good health, and in the morning were surprised to find one or more large vesicles, filled with a clear fluid, upon the above-mentioned parts. After the lapse of a few hours, these vesicles burst, and left a superficial ulcer, which healed in a few weeks, forming a shining, white, circular cicatrix. This vesicular eruption may recur several times during the course of the malady, and in some instances it has not appeared till long after the disease had become fully developed in the constitution. In some cases—but by no means in the majority—an increase of sensibility of the skin has been observed before the opposite state, which has given the distinctive appellation to the disorder, had supervened.

Sooner or later after the above premonitory symptoms, the skin becomes sallow, and often almost of a violet hue ; the integument is dry and loose, and its sensibility greatly diminishes, until, in the fully-developed form, it is entirely lost in the extremities, and also upon the face. In one instance, Dr. Boeck found the whole surface of the body equally destitute of feeling. Animal life may, indeed, be said to be extinguished in the affected portions ; the limbs may be amputated without the least pain to the patient, who often is not aware that the operation has commenced, till he hears the grating of the saw upon the bone ; nor is it rare that these unfortunate individuals, who constantly complain of intense cold, when standing at the heated stoves with their hands behind them, are severely burned in the fingers or legs, without being themselves aware of the injury, till the odour of the burned flesh reaches their nostrils, or till they are apprized of it by their companions.

In this form of elephantiasis the functions of the skin are nearly annihilated, perspiration never is observed, and the integument becomes like half-tanned leather to the touch. But, in some instances, a viscous fluid exudes from the skin, and, concreting, gives rise to vesicles and pustules in the neighbourhood of the parts affected. In such cases the

* The appellation "glabra" is preferred by Dr. Boeck, as he says that the insensibility of the skin, which has given rise to the name of anaisthetos, is sometimes wanting in this form of elephantiasis.

epidermis becomes thickened, and subsequently the entire skin, which then becomes exceedingly prone to a peculiar form of ulceration. A pale, fluctuating, painless spot or point appears in the integument, generally in the soles of the feet; the epidermis splits, and an ichorous fluid is poured out. The ulcer thus formed soon penetrates and undermines the skin, destroying the subcutaneous cellular tissue, and laying the muscles bare; the edges of the sore are sharp and hard, the surface red and dry, with an occasional secretion of an opaque viscous fluid. It is almost impossible to excite suppuration on this surface. Dr. Danielsen has filled the ulcer for many weeks in succession with powdered cantharides, and always failed in producing the slightest suppuration. At times the whole of the soft parts are destroyed, the ligaments and bones are laid bare, and the latter may be destroyed by necrosis, and then thrown off. The tendons of the feet may be thus attacked, the ligaments of the joints bored through, till the joint itself is dislocated, and hangs useless within the integument, while the ulcerated and ruptured tendons protrude through the open sore. Where this destructive process is rapid it is accompanied by great general reaction, but it is more usually chronic in its advances: the patient suffers for a long time previously from pain in the legs, and especially at night, depriving him of sleep; but these nocturnal pains usually cease as soon as the ulcer shows itself upon the surface. Dr. Boeck lays more stress upon these nocturnal pains, and connects them with insidious inflammation and ulceration of the joint, and he also regards the ulcerative process as of a more active kind than Dr. Danielsen represents. But perhaps Dr. Boeck has principally examined those cases where necrosis of the fingers, toes, and metacarpal bones occur with much pain and swelling, and with great general reaction of the system. In such cases the parts about to be affected become hard, red, swollen, and very painful; the tumefaction extends up the limbs towards the groin or the axilla; the glands in these parts inflame, and the whole progress of the malady is marked by much fever, vomiting, headach, and a small and frequent pulse. The original tumour becomes gradually paler, and a fluctuating point is observed in it, which speedily bursts, and a quantity of bloody ichorous fluid is discharged, along with dead cellular tissue. On exploring the ulcer, the necrosed bone is discovered beneath. The general symptoms now decline, the swelling diminishes, and after some time the whole phalanx is thrown off, and the ulcer heals. This process may be frequently repeated in the same subject, until all the fingers or toes are destroyed, and even the metacarpal and metatarsal bones are occasionally lost in this manner.

A species of paralysis of the muscles of the face is noticed by all writers upon the spedalskhed. It produces a peculiarly idiotic cast of countenance; as the orbicularis oris and palpebrarum no longer perform their office, a constant stream of tears furrows the cheeks, while a similar flow of saliva takes place from the dependent corners of the mouth. Ectropion of the lower eyelid frequently occurs, and successive attacks of inflammation, from insufficient covering of the eyeball, at length entirely destroy the functions of that organ.

Contraction of the fingers is also not uncommon, and Dr. Boeck has never seen this annoying symptom yield to any application.

Few of the patients that entered St. George's Hospital in Bergen in 1841 were free from other skin diseases. Many suffered from prurigo, lichen, and eczema at one and the same time; so that the skin of individuals affected with elephantiasis would seem to be a perfect garden for other diseases of the integument. Elephantiasis glabra does not cause the loss of hair from the surface of the body, as is constantly the case in El. tuberculosa; nor does it appear to attack much the internal organs. The uvula is occasionally slightly ulcerated, while the general health of the patient may not be much disturbed, and the secretions, the menstruation, &c. may proceed in their regular course.

The medium age at which El. glabra has been observed to commence is from twenty-three to twenty-five; but from Dr. Boeck's tables we learn, that that malady may commence in the tenderest years, or may not be developed till the patient is in the decline of life. Of the 42 cases observed by Dr. Boeck, 22 were males and 20 females. He assures us that the malady on the coasts of the Mediterranean is identical with that which he has found much more frequent in Norway. In the former localities this form of elephantiasis occurred only in one tenth of the patients; in Norway one third were thus affected.

Second form. *Elephantiasis tuberculosa.* This form of the disease is frequently preceded by a sensation of cold, either general over the whole body, or confined to the part about to become the seat of the malady. Along with this we usually find anorexia, and occasionally an acute febrile paroxysm occurs, accompanied with swelling and redness of the limbs, which last is of so peculiar a character as to indicate to practised eyes the near approach of the disease; but more generally elephantiasis tuberculosa is developed in a gradual manner. The eyelashes, eyebrows, and beard fall off, and sometimes also the hair upon the scalp; but the chief and characteristic symptom are the bluish-red elevations or tubercles, which form in the skin and over all parts of the body, thickening it, and giving to the patient a most repulsive and scarcely human appearance. These tubercles generally appear first upon the face;* the skin of the affected portions soon alters in colour, becoming of a dark bronzed hue, and at the same time it becomes shining, as though it were smeared with oil. If the disease continues to make progress, the tubercles sooner or later soften, and form superficial ulcerations, or else they excrete a thick viscous matter, which hardens into crusts upon the surrounding skin.

After some time the eyes also suffer from the malady. Small elevations, closely resembling the tubercles above described upon the skin, form upon the conjunctiva, and an insidious form of iritis goes on within the eyeball, which often causes the total loss of the sight. The tubercles which form on the conjunctiva commence, without any pain, as a minute yellowish-brown mass, which is surrounded by a small network of bloodvessels. These tubercles are not more than two or three lines in diameter at first, and their general position is on the outside of the cornea. Along with these we find frequently an opacity and thickening of the

* In Rayer's Plates of Diseases of the Skin, there is a drawing of a case of this kind, which exactly resembles one that we saw in Norway, under Dr. Boeck's care at Kongsberg, in 1836.

*conjunctiva corneæ,* which in general obscures only the superior half of the pupil, but may extend over the whole, and totally obstruct vision.

The mucous membrane of the nose swells and ulcerates; at first, the sores are merely superficial, but they often subsequently penetrate the septum narium, and this sometimes is destroyed, and the point of the nose falls down. The hard crusts that form in the nasal cavity from the secretion of these ulcers, is complained of by the patients, as one of the most tormenting symptoms of their disease.

Tubercles also occur in the larynx, but they are seldom of large size, though, from their number, they may seriously interfere with respiration, and the voice is always hoarse, and sometimes almost inaudible. The tonsils, the uvula, and the epiglottis are sometimes nearly destroyed by the ulceration succeeding upon the development of tubercles within their substance. Ulcers in this situation have sharp edges, of a pale red, and the surface of the sore is smooth, and secretes a thick yellowish white fluid.

Occasionally tubercles are discovered in the lungs of patients on dissection; but they are almost always the common pulmonary tubercle, an inorganic, granular, hard, grayish mass, very distinct from the tubercles of elephantiasis, which are fatty and yellowish, tend constantly to ulceration, and bear undoubted marks of being organized products. But though thus rarely seen in the parenchyma of the lungs, they are frequent in the pericardium and in the pleura, and present there characters identical with those found beneath the skin. The abdominal organs may also contain true leprous tubercles, but it is singular how little the appetite or the general health is impaired in such cases, though febrile reaction sometimes is seen at the commencement of the disorder.

So widely different in external character, are the two forms of elephantiasis we have now described, that we should be inclined to consider them as separate maladies, were not both well known to arise from the same causes; which in one individual of a family may give rise to elephantiasis glabra, and in another, under exactly similar circumstances, may occasion the development of the tubercular species. The one, too, often passes into the other, and it is chiefly the elephantiasis tuberculosa that undergoes this transformation. The patient becomes feverish, he complains of a burning sensation in his skin, which becomes red and swollen, and the limbs are peculiarly tender and sensible to the slightest touch. These pains gradually cease, the tubercles disappear, the skin becomes smooth and of a pale yellow hue, and the sensibility of the affected parts diminishes, until all feeling in them is entirely lost.

The spedalskhed is undoubtedly a skin disease in its most perfect form.* The integuments in the tubercular species are totally altered in structure; the skin loses its lamellar structure from the deposition of tubercle or tuberculous matter between the layers, and the whole becomes a homogeneous mass with a few interspersed fibres. Perspiration can hardly be said to exist, while in its place there exudes a thin viscous fluid, which concretes upon exposure to the atmosphere. Nor do the

* Dr. Boeck doubts this assertion, and suggests that the disease cannot be looked upon as one purely of the integument. No doubt many maladies, at present classed as simple skin diseases, will be afterwards proved to arise from an altered condition (dyscrasis) of the blood.

nerves and arteries ramifying in the cellular tissue escape uninjured; the tubercular matter is at first deposited merely upon their circumference, but subsequently it penetrates them entirely, and totally changes their structure; they become homogeneous cords; the nerves no longer contain the cells so characteristic of their nature, and the smaller arterial or venous trunks are totally obliterated, while the muscles usually remain uninjured.

The tubercular form of elephantiasis may occur in any age from infancy to sixty or sixty-five, but it most frequently makes its appearance during the period of manhood.

As to the proportion of the sexes affected by this disease, all agree that it more frequently occurs in males than in females. Of the 90 Norwegian patients whose cases are detailed by Dr. Boeck, there were 47 men. Of the 21 cases seen by him in Italy, 16 were males; and of the 160 observed in Greece, there were only 52 females. The malady everywhere affects those *dwelling on the sea coast.* All the Norwegian male patients were fishermen and sailors, as is universally the case in the Bergen district; and the disorder in Norway, Greece, Italy and France was entirely confined to the lower classes of society. The only difference observed by Dr. Boeck, between the Norwegian disorder, and the form under which it appears in more southern climates, was that the parts of generation were more frequently affected by ulcers and tubercles in the latter, while in Norway, eczema impetiginoides was a very frequent complication.

That the disorder, under both forms, is hereditary, is, we think, amply proved by the elaborate statistical tables of Dr. Boeck; while, like other maladies which are thus transmitted, it appears frequently to pass over a generation or two, and then to recur with increased violence. But the question of its being propagated by contagion is still undetermined. In Bergenstift, the spedalskhed is not looked upon as contagious; the healthy and the afflicted frequently inhabit the same chamber; the married continue their relations, and seek no separation of bed or board, should either party become the subject of the disease; while in the northern parts of Norway, the wretched leper is thrust forth, as of old, from society, and is compelled to drag out a miserable existence in his solitary hut. In Greece, according to Dr. Boeck, the doctrine of contagion is fully received, both by the people and by medical men; nay, even a German physician of eminence, Dr. Roeser, refused to touch Dr. Boeck's hand when he returned from visiting the lepers near Athens, saying that he had practised too long in Greece to remain in doubt of the contagious nature of the disease.

As far as has been hitherto ascertained, the entire number of persons afflicted with the spedalskhed in Norway, amounts to 659; the disease prevails in 1 individual in 511 in Bergenstift; in 1 in 947 in Norland and Finmark, and in 1 in 2505 in Greece. These proportions certainly favour not a little the doctrine of contagion, and the necessity of separating the afflicted from healthy individuals.

It has been maintained that a diet of half-rotten fish and of the oily bodies of sea-fowl is the chief cause of the disease. But the inhabitants of the sea coast in Norway live chiefly upon fish, *either fresh or well salted,* and rarely eat sea-fowl; and when they do so the skin and its accompanying layers of fat are generally removed. Besides, many suffer from the disease, who rarely eat fish or sea-fowl, but who live throughout the year upon flesh-meat, and bread or puddings. The patients themselves invariably ascribe their malady to the daily cold they suffer from wading in the rivers, and among the melting snow in spring, and to the being obliged often to sleep in their wet and frozen clothes. Dr. Boeck thinks that the exhaustion and cold to which the fishermen on the coast are so exposed, contribute greatly to the prevalence of disease in these localities; but all agree that its development is greatly favoured by a damp atmosphere, ill-constructed and ill-ventilated dwellings, the abuse of ardent spirits, and by the filthy habits and total neglect of all personal cleanliness which prevail among the lower classes in Norway.

In France, elephantiasis is to be found upon the southern coast in the neighbourhood of Marseilles, especially in the wretched villages of Vitrol, Rognac, Baer, and Maitigue; but as the malady is there looked upon as a special infliction of Providence, the sufferers carefully conceal themselves from the public view, so much so, that in Vitrol, where the greatest number are to be found, Dr. Boeck could not obtain sight of a single case.

In Italy the disease prevails under the name of "la lebbra," on the coast of Piedmont, and especially in Varaze, a small town near Genoa; but, according to Dr. Granetti, the whole number of the affected does not exceed sixty. Probably the commission just established by the Sardinian government to investigate the disease, will develop a much greater extension of it. On the eastern coast of Italy, elephantiasis also exists, and is there called "male del fegato." In Greece it is known by the name of lova, and is entirely confined to the islands, or to those who dwell on the sea coasts of the continent: the number of sufferers is computed by Dr. Bouros at 160.

*Treatment.* Dr. Boeck's stay in most places that he visited was too short to enable him to follow out the results of any particular plan; we therefore confine ourselves chiefly to the experience of Dr. Danielsen in this respect.

During the year 1841 every possible mode of treatment was tried at St. George's Hospital in Bergen, but, with the exception of two, they were all of no avail. Mercurials gave rise to vomiting and to purging, but never produced salivation, while iodine and the salts of potash were totally unavailing. The preparations of arsenic, consisting of the arseniate of copper, Scheele's green, and Fowler's solution, induced, after a short period, violent reaction in the system, especially inflammatory affections of the bowels, as enteritis and peritonitis, in the presence of which inflammations the tubercles evidently diminished in size, and the thickened skin returned to its normal density; but as soon as the inflammation had subsided the tubercles again increased, and the patient's condition became much worse than before, from the anorexia, vomiting, debility, and flying pains in the bowels, which tormented the sufferers for a long time after. Dr. Danielsen states that he has frequently observed the tubercular masses to disappear, for a time, in the way above described, but they showed themselves again as soon as the acute attack was passed, though upon some fortunate occasions the patient was actually freed from his leprosy by rapidly-succeeding attacks of acute disease. Dr. Danielsen believes in the curability of the malady in its earliest stage; but when once the tubercles have become fully developed, or the paralysed condition of the skin has appeared, then all remedies seem at the most to palliate the disorder. Of all modes of treatment, Dr. Danielsen gives the preference to venesection: it appears, he says, to check that plasticity of the blood which is so characteristic of elephantiasis tuberculosa, and not only is the tendency to tubercular formation diminished thereby, but all the symptoms that precede and accompany it are greatly ameliorated. Dr. Danielsen has found the greatest benefit from venesection, repeated for ten, twelve, or fourteen days in succession; and though the patients were, of course, by such active treatment, greatly reduced in strength, still they often earnestly demanded it to be repeated, from the great alleviation it brought to their sufferings. The blood has always presented a distinct inflammatory crust, of a greenish hue, but no chemical or microscopical investigation of it has hitherto been attempted. As to the elephantiasis glabra, Dr. Danielsen avows that every plan of treatment hitherto attempted has proved totally unavailing.

The result of these commissions of inquiry, established by the governments of the kingdoms where the disease prevails, will no doubt lead to an amelioration and thorough reform of the charitable institutions for the reception of lepers. St. George's Hospital at Bergen scarcely deserves the name: it is a receptacle for the afflicted, who seem to be under no rule or government; and the medicines, as Dr. Danielsen justly complains, are constantly thrown aside or neglected, according to the humour of the patients. It is only by the strictest sanitary regulations that we can expect this terrible disorder to be finally extirpated from every part of Europe.

---

